# Restoring effects of oxytocin on the attentional preference for faces in autism

**DOI:** 10.1038/tp.2017.67

**Published:** 2017-04-18

**Authors:** M Kanat, I Spenthof, A Riedel, L T van Elst, M Heinrichs, G Domes

**Affiliations:** 1 Department of Psychology, Laboratory for Biological and Personality Psychology, University of Freiburg, Freiburg, Germany; 2 Freiburg Brain Imaging Center, University Medical Center, University of Freiburg, Freiburg, Germany; 3 Department of Biological and Clinical Psychology, University of Trier, Trier, Germany; 4 Department of Psychiatry, Section for Experimental Neuropsychiatry, University Medical School Freiburg, Freiburg, Germany

## Abstract

Reduced attentional preference for faces and symptoms of social anxiety are common in autism spectrum disorders (ASDs). The neuropeptide oxytocin triggers anxiolytic functions and enhances eye gaze, facial emotion recognition and neural correlates of face processing in ASD. Here we investigated whether a single dose of oxytocin increases attention to faces in ASD. As a secondary question, we explored the influence of social anxiety on these effects. We tested for oxytocin's effects on attention to neutral faces as compared to houses in a sample of 29 autistic individuals and 30 control participants using a dot-probe paradigm with two different presentation times (100 or 500 ms). A single dose of 24 IU oxytocin was administered in a randomized, double-blind placebo-controlled, cross-over design. Under placebo, ASD individuals paid less attention to faces presented for 500 ms than did controls. Oxytocin administration increased the allocation of attention toward faces in ASD to a level observed in controls. Secondary analyses revealed that these oxytocin effects primarily occurred in ASD individuals with high levels of social anxiety who were characterized by attentional avoidance of faces under placebo. Our results confirm a positive influence of intranasal oxytocin on social attention processes in ASD. Further, they suggest that oxytocin may in particular restore the attentional preference for facial information in ASD individuals with high social anxiety. We conclude that oxytocin's anxiolytic properties may partially account for its positive effects on socio-cognitive functioning in ASD, such as enhanced eye gaze and facial emotion recognition.

## Introduction

Autism spectrum disorders (ASDs) are associated with difficulties in understanding facial cues that potentially arise from reduced attraction by social information in association with a disturbed development of neural face processing networks during early infancy.^1, 2^ For example, autistic children display less attentional orienting but enhanced attentional disengagement from facial cues.^3, 4^ Within the past few years, the neuropeptide oxytocin has been shown to enhance socio-cognitive functions that are frequently impaired in ASDs. For example, intranasal administration of oxytocin improved performance in facial emotion recognition tasks,^5, 6, 7^ increased social trust^8, 9^ and selectively strengthened emotion perceptions for face cues but not for nonsocial information.^10^ These effects may partially rely on oxytocin's modulatory influences on social attention processes, such as enhanced eye gaze^11, 12, 13^ or valence-dependent orienting of attention toward emotional expressions.^12, 14, 15^

Notably, oxytocin effects on attention^16^ and empathic accuracy^17^ were shown to be particularly pronounced in healthy individuals with higher autistic traits. In persons diagnosed with ASD, intranasally administered oxytocin seems to facilitate social interactions and feelings of trust,^18^ promote mentalizing abilities based on facial features^19, 20, 21^ and to increase eye gaze.^11, 18^ In addition, imaging studies demonstrated enhancing effects of oxytocin on functioning of the social brain in ASD, as observed during the processing of face cues^20, 22, 23^ and social judgments,^24, 25^ including the inference of facial emotions.^26^ However, it remains unclear whether oxytocin initially increases social orienting in ASD and thereby potentially corrects for a characteristic impairment that is already observed in autistic children aged 2–5 years.^3, 4^ Beyond this background, the primary hypothesis of our study was that oxytocin enhances attention for social as compared to nonsocial information in ASD. To test these hypotheses, we measured the effects of a single dose of intranasal oxytocin on attention to face and house pictures in the well-established dot-probe task.^27^

Attention for social cues is known to be modulated by anxiety levels. For example, social anxiety has been associated with an attentional bias for negative facial expressions^28^ and attentional avoidance of the eye region, particularly for angry faces.^29^ Symptoms of social anxiety are common in individuals with high-functioning autism and Asperger syndrome, and seem to worsen with age.^30, 31^ Notably, individual levels of social anxiety predict attentional avoidance of the eye region^32^ and intensify impairments in gazing toward a virtual audience in ASD^33^ suggesting that social anxiety may increase social attention deficits as commonly observed in ASD. In contrast, the neuropeptide oxytocin is assumed to reduce symptoms of social anxiety by lowering the responsiveness towards social evaluations^34^ and threat-related facial information.^35, 36, 37^ Imaging studies suggest that oxytocin may attenuate arousal by socially threatening information by modulating activity in the amygdala and associated brain areas.^35, 36, 38, 39^ As a secondary question, we therefore explored whether oxytocin's effects on social attention in autistic individuals might be influenced by social anxiety as measured by a trait questionnaire.^40^

## Materials and methods

### Subjects

A total number of 29 male autistic individuals with Asperger syndrome (ASD group; mean age±s.d.: 38.2±10.6) and 31 neurotypical control subjects (NT group; mean age±s.d.: 31.7±12.3) participated in the study. The sample size was based on previous studies investigating effects of oxytocin on attention processes in ASD.^11, 18^ Women were excluded from participation due to the greater prevalence of ASD in males and to avoid confounding effects of hormonal changes across the menstrual cycle with oxytocin administration. Due to his extreme value in our social anxiety measure, one subject from the neurotypical control sample was excluded from all analyses. Demographic and clinical characteristics of the final sample are described in Table 1. The ASD group scored higher on measures of autism and social anxiety. Both groups were similar in their verbal intelligence and years of education, but the autistic individuals were significantly older than the controls.

Individuals with ASD were mainly out-patients recruited via the Clinic for Psychiatry and Psychotherapy of the University Hospital Freiburg where they had been diagnosed by experienced clinicians. Diagnosis was subsequently validated with Module 4 of the Autism Diagnostic Observation Schedule^41^ by trained members of our research team. This procedure led us to exclude two potential participants, one of whom did not fulfill ASD cutoff criteria, whereas we suspected the other of having cognitive and verbal impairments, leading to the diagnosis of an autistic disorder rather than Asperger syndrome. In addition, comorbidities were assessed by means of self-report. In total, 20 out of 29 participants with ASD (69.0%) reported present or past psychiatric comorbidities, mostly depressive (*n*=18), attention-deficit/hyperactivity (*n*=7), or anxiety disorders (*n*=6; for example, social phobia, panic disorder). The majority of ASD individuals (*n*=18; 62.1%) took psychoactive drugs regularly, including antidepressants and neuroleptics. Neurotypical control subjects were recruited via institutional bulletin boards. They had no physical or psychiatric illness and were not taking any medications as revealed during a thorough interviewing process including the German version of the Structured Clinical Interview for DSM-IV Disorders.^42^ Subjects gave their written informed consent prior to participation and were paid at the end of the last testing session. The study protocol concurred with the Declaration of Helsinki, was approved by the ethics committee of the University Hospital Freiburg and registered in the European Union Clinical Trials Register (EudraCT number 2010-022511-18; URL: https://www.clinicaltrialsregister.eu; registration date: 17 August 2010).

Participants completed a set of standardized questionnaires on demographic, clinical and personality variables. These comprised the German translations of the Social Interaction Anxiety Scale that assesses anxiety symptoms in social interactions,^40^ the Autism-Spectrum Quotient^43^ and the Wortschatztest^44^ as an estimate of verbal intelligence. In addition, participants completed a three-scale state questionnaire immediately before nasal spray administration and prior to the beginning of the experimental task in which they rated mood, wakefulness and arousal.^45^

### Experimental procedures

Participants came for two testing sessions 14 days apart, filling out a questionnaire addressing current medical and psychological conditions at each session. Thereafter, they self-administered three puffs of a nasal spray containing either oxytocin (Syntocinon Spray, Novartis, Basel, Switzerland) or a placebo (containing all ingredients, except the neuropeptide) in each nostril. For the oxytocin spray, this procedure added up to a total dosage of 24 IU. Half of the participants received oxytocin at the first testing session and placebo on the second session in randomized, double-blind fashion. Randomization was done in blocks for 10 participants each by the University Hospital of Heidelberg's pharmacy, which provided the nasal sprays and unblinded the research team on each participant's drug-administration sequence after the completion of data collection. Approximately 45 min following substance administration, participants were seated in front of a computer screen, and completed a dot-probe task that assesses the attentional preference for a target stimulus as compared to a simultaneously presented distractor stimulus based on reaction times to a subsequent probe.^27^

**Figure 1:**
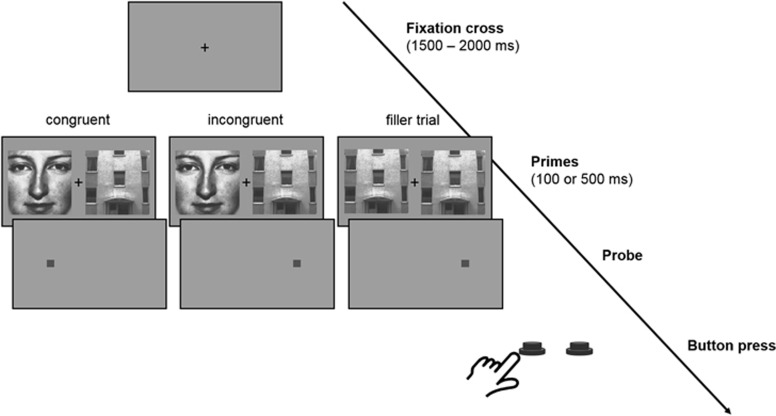
Trial structure and experimental conditions of the house–face dot-probe paradigm.

In the dot-probe task, a set of 12 faces selected from the Karolinska directed emotional faces database^46^ served as social target stimuli. These face pictures were compared to 12 house pictures already used in a another study^22^ that served as nonsocial distractor stimuli. All pictures were converted to gray-scale and had a final size of 200x200 pixels. They were presented against a gray background and displayed on a 22" TFT screen (40.8 × 30.6 cm; 1680x1050 pixels) at a constant viewing distance of 65 cm assured using a chin rest. Trial structure and experimental conditions are depicted in Figure 1. Each trial started with a black fixation cross in the center of the screen for 1500–2000 ms. Then, two pictures were presented simultaneously in the left and right hemisphere at a horizontal distance of 800 pixels and lasting either 100 or 500 ms. Immediately after presentation of the two pictures, the probe (a small gray square, 10x10 pixels) appeared in one of their positions. During the target trials, pictures of a house and a face were simultaneously presented and the probe was cued by a face (congruent) or a house (incongruent) in half of the trials. During the filler trials, either two houses or two faces were displayed. Subjects indicated the location of the probe by pressing one of two buttons as quickly and accurately as possible. The task comprised a total of 96 trials, half of which represented target trials or filler trials. We used Presentation (www.neurobs.com) to control picture presentation and record the number of correct responses and reaction times to the dot probes.

### Statistical analysis

First, we excluded all trials revealing erroneous responses or implausible reaction times (<200 ms or >2500 ms) from further analysis. This accounted for 118 trials (1.0%) of the 11 328 trials presented to the 59 participants. Reaction time (RT) data from valid trials was entered into a repeated measures analysis of variance (ANOVA) with the factors group (ASD, NT), drug condition (placebo, oxytocin), prime duration (100, 500 ms) and probe location (congruent, incongruent). Attentional bias scores were calculated by subtracting the median RT in congruent trials from that in incongruent trials, with positive values reflecting increased attention for the target stimulus (that is, a face). In addition, we accounted for two distinct attention components integrated within the attentional bias, namely the initial allocation of attention to the salient prime (vigilance), and the ability to disengage attention from the prime (adherence), that is, adherence.^47^

The initial allocation of attention to the target stimulus is indicated by faster responses in congruent target trials than in filler trials (ΔRT_allocation_=RT_filler_−RT_congruent_). In contrast, adherence to the target stimulus is revealed by slower responses in incongruent target trials than in filler trials (ΔRT_adherence_=RT_incongruent_−RT_filler_). While we had also included filler trials with faces to prevent faces from having an attentional advantage solely because of a face event's infrequency, only filler trials entailing the simultaneous presentation of two houses were used to calculate allocation and adherence scores.

All three attention scores were analyzed within separate ANOVAs with the factors group (ASD, NT), drug condition (placebo, oxytocin) and prime duration (100, 500 ms). To explore the influence of social anxiety on attention processes in ASD, we calculated correlations between attention measures and individual anxiety levels. Due to a significant correlation within the ASD group under placebo conditions, we subsequently split the ASD group into a low and a highly anxious subsample based on the group median. For the ASD group only, we then calculated a three-way ANOVA with the between-subjects factor social anxiety (low, high), drug condition (placebo, oxytocin) and prime duration (100, 500 ms). All statistical analyses were run in IBM SPSS Statistics 21 (IBM, Armonk, NY, USA) with a statistical significance threshold of *P*<0.05 (two-sided testing). In case the assumption of sphericity was violated, we applied a Greenhouse–Geisser correction.

## Results

### Raw reaction time data

Our initial analysis of raw RT data revealed the probe location's significant main effect, with longer response latencies for incongruent probe locations indicating increased attentional capture by faces as compared to houses (F_1,57_=26.90, *P*<0.001). While ASD individuals were generally slower in responding to the probe (F_1,57_=21.55, *P*<0.001), there was no significant interaction between probe location and group (F_1,57_ =0.35, *P*=0.558) nor between probe location and drug condition (F_1,57_ =0.543, *P*=0.464). Hence, the attentional preference for face stimuli was similar in both groups and independent of oxytocin administration. In contrast, we observed a significant three-way interaction of duration, drug and group (F_1,57_=4.530, *P*=0.038): oxytocin administration tended to reduce response latencies in the NT group for the short stimulus condition (100 ms), and in the ASD group for the long stimulus condition (500 ms). However, within subsequent pairwise analyses, none of these effects reached significance (see Table 2 for raw reaction time data).

### Attentional bias

We next analyzed group differences for the attentional bias (that is, the difference between RTs for incongruent versus congruent probe locations) and its modulations by prime duration and oxytocin administration. The corresponding three-way ANOVA yielded no significant results, neither for the main effects nor any of the interactions. Thus, the attentional preference for face stimuli was similar in ASD individuals and NT controls, and not modulated by prime duration or oxytocin administration.

### Allocation versus adherence

Within separate ANOVAs, we tested for effects on vigilance, that is, the initial allocation of attention toward faces, and adherence, that is, difficulty disengaging attention from faces.

We observed no significant group differences, effects of prime duration or interactions between the two under placebo conditions in the adherence scores (all *P*>0.05). A subsequent three-way ANOVA with the additional factor drug condition revealed no significant effects of oxytocin administration on adherence (all *P*>0.05), indicating that the difficulty in disengaging attention from faces was not influenced by group, prime duration or drug condition.

**Figure 2:**
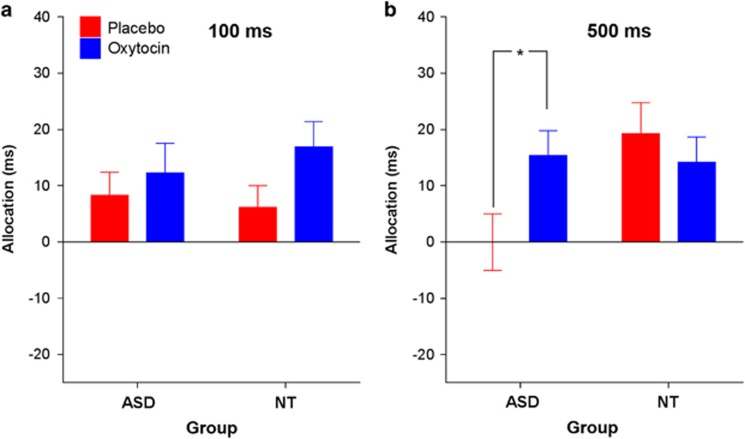
Effects of intranasal oxytocin on allocation of attention to faces versus houses presented with (**a**) short duration (100 ms) or (**b**) long duration (500 ms). Error bars represent the s.e.m. *t_28_=−2.63, *P*=0.014. ASD, autism spectrum disorder; NT, neurotypical.

Regarding the allocation scores, a two-way ANOVA with the factors group and prime duration revealed a significant interaction between both factors under placebo conditions (F_1,57_=6.10, *P*=0.017): individuals with ASD and the controls both allocated attention similarly to faces in the brief stimulus condition (*t*_57_ =0.38, *P*=0.704) whereas the ASD group exhibited a lower initial allocation of attention to face stimuli in the long stimulus condition (*t*_57_=−2.58, *P*=0.012). In a subsequent three-way ANOVA with the additional factor drug condition, we noted a main effect of drug condition on the initial allocation of attention to faces: oxytocin administration generally increased the allocation of attention to faces as compared to houses (F_1,57_=4.27, *P*=0.043). This effect was further modulated by group and prime duration, as indicated by a significant three-way interaction (F_1,57_=4.10, *P*=0.048) (Figure 2). Pairwise comparisons showed that oxytocin tended to enhance attention to faces in the NT group for the short stimulus condition (*t*_29_=−1.97, *P*=0.059). In contrast, oxytocin significantly enhanced attention to faces as compared to houses in the ASD group for presentations of 500 ms (*t*_28_=−2.63, *P*=0.014), thereby eliminating the impaired social attention observed under placebo conditions.

### The influence of social anxiety on the allocation of attention to faces

**Figure 3:**
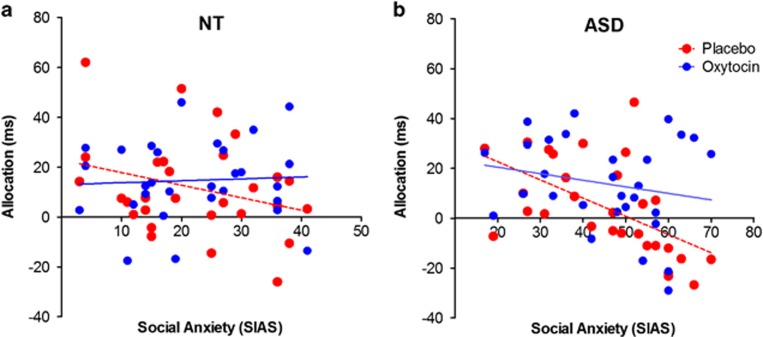
Association between the allocation of attention to faces in the dot-probe task and social anxiety in both groups and its modulation by oxytocin administration. (**a**) In the neurotypical control group, social anxiety showed a weak negative association with attention to faces under placebo (*r*=−0.30, *P*=0.119), which vanished following oxytocin administration (*r* =0.05, *P*=0.785). (**b**) In the autistic group, social anxiety significantly predicted attentional avoidance of faces under placebo (*r*=−0.56, *P*<0.05), whereas this association was no longer significant under oxytocin (*r*=−0.20, *P*=0.289). ASD, autism spectrum disorder; NT, neurotypical; SIAS, Social Interaction Anxiety Scale.

Within secondary analyses, we next explored whether social anxiety levels influenced the initial allocation of attention to faces. In the ASD group, allocation of attention to faces showed a significant negative correlation with individual levels of social anxiety under placebo conditions, indicating reduced attention to faces in highly anxious persons (ASD_Placebo_: *r*=−0.56, *P*=0.002) (Figure 3). We observed a similar correlation within the NT group, but it failed to reach statistical significance (NT_Placebo_: *r*=−0.30, *P*=0.119). Following oxytocin administration, the negative association between social anxiety and attention to faces was reduced in the ASD group (ASD_Oxytocin_: *r*=−0.20, *P*=0.289) and vanished in the NT group (NT_Oxytocin_: *r* =0.05, *P*=0.785). The difference between the two correlations under placebo and oxytocin conditions approached significance in both groups (ASD: *z*=−1.50, *P*=0.07; NT: *z*=−1.61, *P*=0.05).

We subsequently included social anxiety as a covariate in our three-way analysis of variance on allocation scores (factors group, prime duration and drug condition). This analysis yielded a significant effect of anxiety on allocation scores (F_1,55_=8.46, *P*=0.005) as well as a significant interaction between anxiety and oxytocin administration (F_1,55_=4.68, *P*=0.035). Of note, neither the above-reported main effect of oxytocin administration nor the interaction between drug condition, stimulus duration and group continued to be significant (all *P*>0.05). Separate analyses in both groups showed a significant effect of social anxiety in the ASD group (F_1__,27_=10.51, *P*=0.003) but no effects of or interactions with oxytocin administration (all *P*>0.05). The NT group exhibited no significant effects (all *P*>0.05).

**Figure 4:**
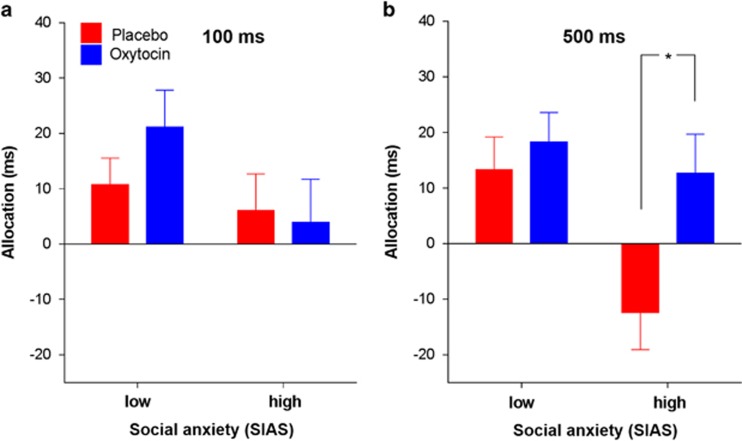
Influence of high and low social anxiety on oxytocin's effect on the ASD group's allocation of attention toward faces versus houses presented with (**a**) short duration (100 ms) or (**b**) long duration (500 ms). Error bars represent the s.e.m. *t_14_=−2.92, *P*=0.011. ASD, autism spectrum disorder; SIAS, Social Interaction Anxiety Scale.

To elucidate the modulating role of social anxiety in the ASD group, we subsequently split the sample by median, thereby differentiating between individuals with high and low levels of social anxiety. In a three-way ANOVA with the factors drug condition, prime duration and the group factor social anxiety, a trend for a three-way interaction emerged (F_1,27_=3.58, *P*=0.069) (Figure 4). Pairwise comparisons of this interaction proved that oxytocin selectively influenced the allocation of attention to faces in highly anxious ASD subjects in the long stimulus condition: A single dose of oxytocin induced attentional orienting toward face stimuli and thereby reversed attentional avoidance as suggested by negative allocation scores under placebo conditions (*t*_14_=−2.92, *P*=0.011). In contrast, no other comparison attained significance (all *P*>0.05).

## Discussion

Our study delivers initial evidence that the neuropeptide oxytocin may enhance visual attention toward face cues in ASD to a level observed in neurotypical controls. These positive effects of oxytocin on social attention seem to primarily occur in ASD individuals characterized by high levels of social anxiety and concomitant attentional avoidance of faces.

The absence of an overall attentional bias toward target stimuli (that is, faces) in our study may be due to the fact that attentional bias integrates two distinct attention components, namely the initial allocation of attention and the adherence of attention to a target stimulus, which might negate each other.^47^ By differentiating between these components, we found that individuals with ASD were impaired in vigilance, that is, the initial allocation of attention toward faces, but demonstrated normal disengagement from facial information. Our results concur with eye-tracking studies reporting a slower initial orientation toward social information in autistic samples as reflected by longer latency to first fixate social cues.^48, 49^ While we did not assess eye movements in our study, we observed impairments in the initial allocation of attention to social cues in ASD for presentations lasting 500 ms, that is, at a stage of stimulus processing that likely matched early gazes to one of the two pictures. In contrast, we observed no attentional differences between ASD and neurotypical individuals in conjunction with 100 ms presentation times, which refer to covert attentional processes. Alterations in social attention in ASD may therefore relate to an early stage of attentional processing, namely the first overt attention shift following conscious stimulus perception.^50^

It has been proposed that decreased attention to faces in ASD may either stem from insufficient emotional salience and motivational indifference toward these cues, or from avoidance behavior as a strategy to regulate heightened arousal.^51^ In our study, exploratory analyses revealed that the initial allocation of attention to faces was much weaker in ASD individuals with high than with low levels of social anxiety, potentially indicating arousal-related attentional avoidance rather than reduced stimulus salience in this subgroup. Notably, we also observed a negative association between social anxiety and the initial allocation of attention toward faces in the neurotypical control sample that did not reach statistical significance, which may partially be attributed to less variance in social anxiety levels in this group. However, it seems plausible that social anxiety's influence on social attention processes may differ in autistic and neurotypical populations. In particular, social anxiety may increase arousal and compensatory avoidance behavior during face processing, especially in individuals with deficits in interpreting social signals.

Our findings are in line with a previous study in which social anxiety increased the avoidance of faces in a simulated public speaking task in children with ASD.^33^ It also supplements findings from a recent study in which participants with ASD displayed reduced attention to videos with actors as compared to videos with objects only under conditions in which the target seemed to move toward them.^52^ In contrast, two dot-probe studies detected no relationship between attentional biases for emotional faces and anxiety symptoms in autistic subjects.^53, 54^ However, those studies assessed multiple dimensions of anxiety including separation anxiety or agoraphobia, and not specifically social anxiety. Moreover, social anxiety in ASD may evoke general avoidance of face cues rather than attentional bias toward specific facial expressions; the latter may additionally be confounded by deficits in emotion recognition in ASD.

The enhancing effect of oxytocin on the allocation of attention to faces in ASD complements a previous study in which oxytocin increased attentional bias as well as recognition accuracy for (positive and neutral) faces but not for nonsocial items (numbers) during a rapid series visual presentation task in healthy individuals with heightened autistic traits.^16^ Together, these findings support the assumption that oxytocin may selectively increase the salience of social information in individuals with low socio-cognitive abilities.^17^ As a novel finding, the improvement in social attention in ASD by oxytocin was influenced by individual levels of social anxiety. Enhancing effects of oxytocin on attention to faces primarily emerged in autistic individuals with high levels of social anxiety who displayed an attentional disregard of face stimuli under placebo conditions. This finding raises the question whether oxytocin may enhance social attention in ASD as a function of social anxiety by decreasing perceptions of social threat and associated arousal. Such a mechanism would concur with meta-analytic evidence that oxytocin particularly reduces stress responses under conditions of strong HPA axis activation, and in clinical populations with social impairments.^55^

Social anxiety has been associated with early hypervigilance for social threat cues (for example, the eye region of faces, aversively conditioned faces) followed by attentional avoidance of the same stimuli at a later stage of stimulus processing.^56, 57, 58^ Our findings seem to mirror this pattern, as we observed attentional avoidance of faces specifically in conjunction with the longer stimulus duration that allowed for overt attention shifts. This suggests that oxytocin might counteract the effortful withdrawal of attention from social cues in anxious individuals with ASD. However, our data does not clarify whether this effect is specific to ASD individuals or is simply driven by anxiety, but was absent in the control group due to its fewer subjects who scored high on social anxiety.

The assumption that oxytocin's anxiolytic and stress-reducing properties might contribute to its positive effects on social attention in socially anxious ASD individuals also concurs with oxytocin's normalizing effects on attentional biases^59^ and amygdala responses^36^ to emotional faces in social anxiety. In autistic individuals, oxytocin administration was found to enhance eye gaze^11, 60^ which is typically impaired in ASD as a function of reduced attentional orienting and active avoidance of the eye region.^61^ Notably, attentional avoidance of the eye region increases with social anxiety^32^ and is predictive of emotion recognition deficits in ASD.^32, 61^ In sum, our findings deliver preliminary evidence that oxytocin may help individuals with ASD to overcome the fear of orienting attention toward a face in the first instance. As this represents a central precondition for finer-grained face scanning, it may underlie oxytocin's positive effects on eye gaze and facial emotion recognition in ASD.

Evidence from imaging studies suggests that altered face processing in ASD is associated with atypical amygdala responses^62, 63, 64^ and that oxytocin modulates amygdala responses during face processing in typically developed persons^65^ and in those with ASD.^20, 22^ For instance, oxytocin has recently been shown to enhance amygdala functioning during face processing in individuals with ASD.^20, 22^ As this enhancement was associated with improved emotion recognition performance, it was interpreted as an increase in social salience rather than emotional arousal.^20^ However, Kleinhans *et al.*^51^ note that findings on altered amygdala functioning in ASD are inconsistent. They propose that attentional disregard due to reduced salience of social information may be associated with the amygdala's hypoactivation. In contrast, active avoidance of social stimuli may serve to prevent emotional overarousal in socially anxious individuals, as reflected in a hyper-reactive amygdala.^51^ Indeed, social anxiety predicted amygdala activation to threatening facial expressions in ASD.^51^ Hence, oxytocin may influence amygdala reactivity to facial expression in ASD differentially depending on individual clinical characteristics along the dimensions of social anxiety and stimulus aversiveness.

If the oxytocin-induced increase in attention to faces in our study was indeed mediated by reduced social anxiety, this should be associated with lower amygdala responses, which would be in line with weakening oxytocin effects on amygdala responses to threat-related facial information in social anxiety disorder and borderline personality disorder.^35, 36^ However, in healthy populations, oxytocin exerts differential effects on amygdala activation during attentional orienting and valence processing.^66^ Future studies should therefore attempt to disentangle the effects of oxytocin on amygdala responses during attention- and arousal-related processes in ASD individuals with low and high levels of social anxiety. Furthermore, it would be interesting to explore whether an age-dependent increase in social anxiety in ASD^30^ may partially explain why the few randomized clinical trials on the therapeutic potential of oxytocin in ASD observed beneficial effects of oxytocin mainly in adult populations.^19, 67, 68, 69, 70^

It should be noted that, to our knowledge, our study was first and foremost designed to test for oxytocin's general effects on social attention in ASD, and not for any moderation or even mediation of these effects by anxiety measures. Therefore, our findings should be considered preliminary and interpreted with caution. Even though our autistic and neurotypical samples both showed substantial variance in social anxiety and differed significantly in this measure, a more straight-forward way to account for the influence of trait anxiety in future studies might imply stratified purposeful sampling techniques or the inclusion of a socially anxious control group without autism. Moreover, although we observed no oxytocin effects on state measures of mood and arousal in our study, it may be worthwhile in future studies to test explicitly for modulations in stimulus-related arousal and state anxiety via oxytocin in the context of social attention.

Taken together, the present study demonstrates that oxytocin enhances social attention in ASD and that these effects are influenced by social anxiety. Our results reveal that oxytocin may in particular normalize effortful attentional avoidance of facial information in ASD individuals with high social anxiety. It may thereby facilitate more elaborate processing of facial cues in autistic persons and improve facial emotion recognition as previously reported. Future studies should investigate whether oxytocin's modulatory effects on eye gaze and amygdala functioning in ASD are attributable to anxiolytic mechanisms, or are better explained by generally enhanced orienting toward social cues.

## Figures and Tables

**Table 1:** Demographic and clinical characteristics of the study groups

	*ASD* *(*n=*29)*	*NT* *(*n=*30)*	*Statistical test*
	*Mean (s.d.)*	*Mean (s.d.)*	
Age (years)	38.2 (10.6)	32.1 (12.3)	*T*_57_=2.04, *P*=0.046
Years in school	13.4 (1.7)	12.9 (0.9)	*T*_40.49_=1.27, *P*=0.212
Verbal intelligence (WST)	34.3 (5.0)	34.2 (3.0)	*T*_56_=0.11, *P*=0.911
Autistic symptoms (AQ)	37.8 (8.1)	18.9 (6.8)	*T*_56_=9.68, *P*<0.001
Social anxiety (SIAS)	45.4 (14.4)	22.0 (11.1)	*T*_56_=6.91, *P*<0.001

Abbreviations: AQ, Autism Spectrum Quotient; ASD, autism spectrum disorder; NT, neurotypical; SIAS, Social Interaction Anxiety Scale; WST, Wortschatztest.

**Table 2:** Average raw reaction times (in ms) for the different conditions in the house–face dot-probe paradigm

	*ASD* *(*n=*29)*		*NT* *(*n=*30)*	
	*Placebo*	*Oxytocin*	*Placebo*	*Oxytocin*
	*Median (s.d.)*	*Median (s.d.)*	*Median (s.d.)*	*Median (s.d.)*
*100 ms*				
Trials congruent	463.1 (57.4)	462.1 (50.6)	407.8 (41.1)	397.8 (46.3)
Trials incongruent	472.9 (71.2)	467.9 (58.1)	415.2 (41.2)	408.3 (39.9)
Filler house	471.4 (53.2)	474.4 (52.2)	413.9 (37.8)	414.7 (44.5)
				
*500 ms*				
Trials congruent	462.3 (68.6)	444.3 (55.8)	399.2 (38.7)	398.5 (44.3)
Trials incongruent	467.8 (65.5)	461.6 (67.6)	414.4 (47.7)	413.6 (46.1)
Filler house	462.3 (64.3)	459.7 (60.3)	418.5 (38.4)	412.6 (43.1)

Abbreviations: ASD, autism spectrum disorder; NT, neurotypical.
